# Neural dynamics of perceptual detection under temporal uncertainty

**DOI:** 10.1186/1471-2202-15-S1-P70

**Published:** 2014-07-21

**Authors:** Federico Carnevale, Omri Barak, Victor de Lafuente, Ranulfo Romo, Nestor Parga

**Affiliations:** 1 Departmento de Física Teórica, Universidad Autónoma de Madrid, Cantoblanco 28049, Madrid, Spain; 2 Faculty of Medicine, Technion - Israel Institute of Technology, Haifa 32000, Israel; 3 Instituto de Neurobiología, Universidad Nacional Autónoma de México, Querétaro 76230, México; 4 El Colegio Nacional, 06020 México DF, México; 5 Instituto de Fisiología Celular, Universidad Nacional Autónoma de México, 04510 México DF, México

## 

Perceptual detection requires the extraction of information about the world from noisy sensory signals. Under uncertainty, the brain uses previous knowledge to transform the sensory inputs into the percepts on which decisions are based. How is this achieved when the uncertainty lies in the timing of the task? How do neural circuits make use of temporal information acquired during learning to solve a perceptual decision-making task?

We study this issue using a recurrent network model and data recorded in monkeys performing a vibrotactile detection task [1]. Importantly, the time of stimulation was variable but confined to an uncued temporal window [2]. We hypothesized that this variability leads to a dynamic response threshold, reflecting the animal’s previous knowledge about the task's temporal structure. Here we aim to find neural signatures of this threshold and study possible dynamical mechanisms underlying perceptual detection under temporal uncertainty.

Previous knowledge about the task should be reflected in the probability of erroneously reporting a stimulus in stimulus-absent trials (false alarms). We found that premotor cortex neurons' activity during single false alarm trials shows localized fluctuations that resemble their activity under stimulation. We hypothesize that these fluctuations are signatures of false alarms. We devised a procedure to extract the times of these events and estimated the probability of false alarms over time. The resulting probability is not uniform but increases in agreement with the probability of stimulation.

We performed a state-space analysis [3] to study the dynamics of perceptual detection at a population level. Projecting the average population activity onto two task-related axes (stimulus and choice), we found that the neural trajectory in correct rejection trials is modulated during the period of possible stimulation, suggesting the use of knowledge about the task’s temporal structure.

What mechanisms support detection under temporal uncertainty? We trained an initially random recurrent neural network to answer this question [4]. Importantly, the teaching signal used during training was restricted to the behavioral outcome and did not convey any information about the probability of stimulation over time. The resulting network learns to perform the task. Furthermore, it is able to infer the task's temporal structure. The lowest stimulus amplitude that drives the network to a stimulus-present decision decreases during the period of possible stimulation, resulting in a probability of false alarms consistent with the one found in the experimental data. Reverse-engineering the model [5] reveals that the decision is dynamically implemented by a saddle point that defines a separatrix between two attractors. The knowledge acquired during training about the task’s timing is encoded in the distance to that separatrix.
